# Effect of different types of olive oil pomace dietary supplementation on the rumen microbial community profile in Comisana ewes

**DOI:** 10.1038/s41598-018-26713-w

**Published:** 2018-05-31

**Authors:** Federica Mannelli, Alice Cappucci, Francesco Pini, Roberta Pastorelli, Francesca Decorosi, Luciana Giovannetti, Marcello Mele, Sara Minieri, Giuseppe Conte, Mariano Pauselli, Stefano Rapaccini, Carlo Viti, Arianna Buccioni

**Affiliations:** 1 Dipartimento di Scienze delle Produzioni Agro-alimentari e dell’Ambiente, University of Florence, Piazzale delle Cascine 18, I-50144 Firenze, Italy; 2 Dipartimento di Scienze Agrarie, Alimentari e Agro-ambientali, University of Pisa, via del Borghetto 80, I-56124 Pisa, Italy; 3 CREA-AA, Consiglio per la Ricerca in Agricoltura e l’Analisi dell’Economia Agraria, Research Centre for Agriculture and Environment, via di Lanciola 12/A, I-50125 Cascine del Riccio, Florence Italy; 4 Dipartimento di Scienze Veterinarie, University of Pisa, Viale delle Piagge 2, 56124 Pisa, Italy; 5 Dipartimento di Scienze Agrarie Alimentari ed Ambientali, University of Perugia, Borgo XX Giugno 74, I-06121 Perugia, Italy

## Abstract

Olive oil pomace (OOP) is a bio-waste rich in highly soluble polyphenols. OOP has been proposed as an additive in ruminant feeding to modulate rumen fermentations. Three groups of ewes were fed the following different diets: a control diet and two diets supplemented with OOP, obtained with a two-phase (OOP2) or three-phase (OOP3) olive milling process. Rumen liquor (RL) showed a higher content of 18:3 *cis*9 *cis*12 *cis*15 (α-linolenic acid, α-LNA) with OOP2 inclusion, and of 18:2 *cis*9 *trans*11 (rumenic acid, RA) with OOP3 inclusion. The overall composition of the RL microbiota did not differ among treatments. Significant differences, between control and treated groups, were found for six bacterial *taxa*. In particular, RL microbiota from animals fed OOPs showed a reduction in *Anaerovibrio*, a lipase-producing bacterium. The decrease in the *Anaerovibrio* genus may lead to a reduction in lipolysis, thus lowering the amount of polyunsaturated fatty acids available for biohydrogenation. Milk from animals fed OOP showed a higher content of 18:1 *cis*9 (oleic acid, OA) but the α-LNA concentration was increased in milk from animals treated with OOP2 only. Therefore, inclusion of OOP in ruminant diets may be a tool to ameliorate the nutritional characteristics of milk.

## Introduction

Increasing the content of polyunsaturated fatty acids (PUFAs) in ruminant derived products is particularly important for human health (i.e., decrease in plasma cholesterol and low-density lipoprotein-cholesterol). Different approaches are used to achieve this result, including the use of diet supplements, such as vegetable oils and other natural compounds, to alter the rumen microbiota^1^. Adding extruded linseed to ewe diets approximately doubles the contents of 18:2 *cis*9 *trans*11 (rumenic acid, RA), 18:1 *trans*11 (vaccenic acid, VA) and 18:3 *cis*9 *cis*12 *cis*15 (α-linolenic acid, α-LNA) acids in milk^2^. The combination of extruded linseed with natural bioactive compounds may further boost milk quality^3,4^. Olive oil pomace (OOP) is the main by-product of olive oil manufacturing and constitutes an important source of nutraceutical molecules with antioxidant and antimicrobial activities, including polyphenols (flavonoids, anthocyans, cyanidins and phenolic acids), tyrosol, hydroxytyrosol and oleuropein^5–8^. OOP requires specific management and storage because it cannot be directly disposed of in the environment^9^. The chemical characteristic of this bio-waste could be valorized by ruminant metabolism^10^. Indeed, several studies have demonstrated that dietary supplementation with OOP at low concentrations in small ruminant diets increases the yield and nutritional quality of milk without having a negative influence on animal welfare, as ewes have a higher sensitivity to polyphenols than goats^11,12^. In an *in vitro* trial, Pallara *et al*.^13^ demonstrated that OOP affects rumen biohydrogenation (BH) of PUFAs, especially linoleic acid (18:2 *cis*9 *cis*12; LA). Other matrices rich in polyphenols, such as tannins from wine peels, chestnut wood or quebracho seeds, are already used in ruminant feeding strategies to improve milk and meat quality with significant results^3,4,14,15^. OOP is usually produced with a three-phase (OOP3) or two-phase (OOP2) decanter. These two bio-wastes differ in their chemical and physical properties, and OOP2 is richer in polyphenols because phenol washing is limited^16^. It is hypothesized that inclusion of the two OOPs in a diet may have similar but not identical effects on the rumen microbiota and the BH process. Hence, this study aimed to investigate the effects of these two different OOPs added to ewe diets on rumen liquor (RL) microbiota, RL fatty acid (FA) and the milk FA profile.

## Methods

### Experimental design

Twenty-four multiparous Comisana ewes at 97 ± 12 days in milking (kept with the experimental flock of the Department of Agriculture, Food and Environmental Sciences at University of Perugia) were allotted into 3 experimental groups (8 animals per pen), with similar body weight (65 ± 8 kg) and milk yield (735 ± 15 g/day). The trial lasted 28 days, after 15 days of adaptation to the new diets.

### OOP characterization

The OOPs used as supplementation in this trial were obtained from local virgin olive oil producers, processed according to Servili *et al*.^17^ and pitted. OOP2 was derived from a two-phase milling process, resulting in a considerable percentage of water (approximately 75%). To make this matrix more technologically suitable, the pomace was adsorbed on ground dried alfalfa to be pelleted with the other ingredients of the concentrate (CMS-IEM – Colognola ai Colli, Verona, Italy). OOP3 was derived from a three-phase process resulting in a low content of water (approximately 55%). The total amount and characterization of OOP polyphenols in the experimental diets were determined by HPLC analysis according to Mele *et al*.^18^, (Supplementary Tables S1 and S2).

### Diet composition

Diets were composed of chopped alfalfa hay (particle size >3 cm in length) administered *ad libitum* with 800 g/head/day of a concentrate formulated to contain the same amount of OOPs as follows: 10 g/100 g of dry matter (DM) of extruded linseed as an α-LNA source (control, diet C), 10 g/100 g of DM of extruded linseed and 13.5 g/100 g of DM of OOP2 (diet COOP2), or 10 g/100 g on DM of extruded linseed and 11.25 g/100 g of DM of OOP3 (diet COOP3) with 100 g/head/day of rolled barley. All concentrates were obtained by pelleting the ingredients (diameter was 5 mm) and offered in two equal doses with rolled barley, during each milking at 7:30 a.m. and 5:30 p.m. (Supplementary Tables S1 and S2). The experimental diets were formulated to be isoproteic and isoenergetic according to the nutrient requirements of an ewe weighing 68 kg and producing 1 kg of milk at 6.5% fat^19^. Animals had free access to water. Dry matter intake (DMI) of concentrates and hay was registered daily and individually on the basis of residuals.

### Rumen liquor (RL) sampling and fatty acid (FA) and dimethyl acetal (DMA) determination

At day 28, RL samples were individually collected by a stomach tube, connected to a manual pump, after overnight fasting and before morning feeding^20,21^. Animals were fasted before rumen sampling to facilitate the introduction of the stomach tube in the rumen as common in the veterinary practice. Five samples from each animal were collected and examined visually and tactilely to check the presence of saliva contamination. Samples from each animal were then combined, strained through a cheesecloth and allotted (20 ml)^20^. Two ewes (one belonging to the C group and one belonging to the COOP3 group) were not considered at sampling time due to diseases. Immediately after collection, each sample was measured for pH, divided into 2 aliquots and stored at −80 °C until analysis. One aliquot was freeze-dried and used for FA and DMA identification according to Alves *et al*.^22^. DMAs are secondary artifacts formed during the methylation of microbial fatty acid methyl esters (FAMEs) derived from bacterial plasmalogen lipids contained in the external membrane and, hence, strictly related to microbial species^22^. First, FAs were trans-esterified^22,23^ using 5:0 and 19:0 (1 mg/ml) as internal standards. FA and DMA fractions were then separated by thin-layer chromatography (TLC). The DMAs were identified by GC/MS^22^, while the FAME profile was determined using a GC2010 Shimadzu gas chromatograph (Shimadzu, Columbia, MD), equipped with a flame-ionization detector and a high-polarity fused-silica capillary column (Chrompack CP-Sil 88 Varian, Middelburg, the Netherlands; 100 m, 0.25 mm i.d.; film thickness of 0.20 μm). The programming used has been previously described by Buccioni *et al*.^3^ (specifications are also available in the supplementary information).

### DNA extraction, PCR amplification, illumina MiSeq sequencing and sequencing data processing

DNA was extracted from 1 ml of RL using a Fast DNA Spin kit for soil (MP Biomedicals, Solon, OH) with the following modifications: 1 ml of RL was thawed and vortexed for 30 s; 185 μl of RL was then mixed lysis buffer, and the mixture was then added to a tube containing the lysis matrix and homogenized with a Retsch MM300 disrupter (90 s at 30 cycles/s). Samples were incubated for 20 min at 70 °C and centrifuged at 14,000 × *g* at 4 °C. The supernatant was recovered and processed according to the manufacturer’s specification. DNA integrity was verified by agarose gel electrophoresis. DNA purity and quantity were measured using a ND-1000 Spectrophotometer (NanoDrop Technologies, Labtech, Ringmer, UK) and standardized to a concentration of 10 ng/μl. For each sample, the V3-V4 region of the 16S rRNA gene was amplified with Pro341f and Pro805R primers^24^, and barcodes were added to the forward primer (Supplementary Table S3). Amplicons for each library were purified and mixed in equal proportions. Illumina MiSeq v3 chemistry 300 base paired-end (PE) sequencing was performed at BMR Genomics (Padova, Italy). MiSeq 300 PE sequencing produced a total of 3,983,079 reads. Reads were merged with FLASh v1.2.11^25^ with the following parameters: -m 20, -M 280, and Phred score default of 33, resulting in 3,362,386 reads correctly aligned reads. The sequences were then trimmed to discard primers with Prinseq-lite^26^, and sequences shorter than 200 bp were filtered out. Chimeras were removed with USEARCH 6.1^27^. Open reference OTU picking was performed with SUMACLUST within QIIME 1.9.1^28^ using a similarity threshold of 0.97 and Greengenes 13.8^29^ as a reference database. OTUs representing less than 0.005% of the total read abundance were discarded^30^. Sequences identified as chloroplasts, mitochondria and unassigned sequences (approximately 5% of sequences in each library) were removed from further analysis. A total of 1,003,318 high-quality sequences were obtained with an average of 45,605 ± 12,742 sequences per sample, and libraries were then rarefied to 30,000 sequences per sample. QIIME tables at different taxonomic levels are available in Supplementary Tables S4–S8.

### Milk sampling and analysis

Individual milk samples were collected weekly, during the morning and evening milking. Milk samples were gathered in a single sample according to the morning and afternoon yield and subsequently split into two aliquots for analysis. The first aliquot was processed to evaluate fat, lactose, protein and urea contents using a Milkoscan 6000 FT (Foss Electric, Hillerød, Denmark) and to determine the somatic cell count (SCC) according to ISO 13366-2/IDF 148-2 (ISO-IDF, 2006) using a Fossmatic 5000 (Foss Electric). Somatic cell count data were expressed as a linear score (LS) according to Shook *et al*.^31^ as follows: LS = log2 (SCC/12,500). Milk production was standardized as fat-corrected milk (FCM) at 6.5% fat according to Pulina and Nudda^32^. The second milk sample aliquots were analyzed for FA composition. Milk fat was extracted as reported by Buccioni *et al*.^3^, methylated according to Christie^33^ with nonanoic (C9:0) and nonadecanoic (C19:0) acid methyl ester (Sigma Chemical Co., St. Louis, MO) as the internal standards and analyzed by gas chromatography using the same program as described for RL samples.

### Statistical analysis

Data on RL FA and DMA were analyzed by the following general linear model:

1 $${{\rm{y}}}_{{\rm{i}}}{\rm{=}}{\mu }{\rm{+}}{\rm{d}}{\rm{i}}{\rm{e}}{{\rm{t}}}_{{\rm{i}}}+{{\rm{e}}}_{{\rm{i}}{\rm{j}}}$$

where y is the observation, *μ* is the overall mean, diet is the fixed effect of i^th^ diet (i = 1 to 3), and e_ij_ is the residual error (SAS 9.2, 2013)^34^.

Data related to animal performances, milk composition and yield recorded over the course of the trial were processed as a completely randomized design with repeated measures using the following linear mixed model (SAS 9.2, 2013)^34^:

2 $${{\rm{y}}}_{{\rm{ijk}}}=\mu +{{\rm{D}}}_{{\rm{i}}}+{{\rm{P}}}_{{\rm{j}}}+{({\rm{D}}\times {\rm{P}})}_{{\rm{ij}}}+{{\rm{A}}}_{{\rm{k}}}[{{\rm{D}}}_{{\rm{i}}}]+{{\rm{e}}}_{{\rm{ijk}}}$$

where y is the observation, *μ* is the overall mean, D_i_ is the fixed effect of diet (i = 1 to 3), P_j_ is the fixed effect of sampling time (j = 1 to 4), (D × P)_ij_ is the interaction between diet and sampling time, A_k_ is the random effect of the animal nested within the diet (k = 1 to 8), and e_ijk_ is the residual error. The covariance structure was compound symmetry, which was selected based on Akaike’s information criterion of the mixed model of SAS^34^. Statistical significance of the diet effect was tested against variance of ewe nested within diet according to repeated measures design theory^35^. Multiple comparisons among means were performed using the Tukey test^34^.

Rarefaction analysis was performed using observed OTUs with 10 iterations at each sampling depth. α-diversity was estimated using observed OTUs, Chao1 value and Shannon index within QIIME. Effects of different diets on FA concentrations, and the relative abundances of different *taxa* were analyzed using a one-way ANOVA with Tukey’s HSD post hoc comparison procedure available within the *agricolae* package in R^36^. A non-metric multi-dimensional scaling (nMDS) plot was constructed using the OTU table with the Bray-Curtis index within PAST^37^. Microbial community profiles were further evaluated with multivariate statistical tests within PAST^37^: One-way analysis of similarity (ANOSIM) and permutational multivariate analysis of variance (PERMANOVA) were performed using Bray-Curtis and Dice indexes (9,999 permutation test).

Pairwise correlation among bacterial *taxa* and FA or DMA composition was performed out by multivariate analysis (SAS, 9.2, 2013).

### Use of experimental animals

All experiments in this study were performed in accordance with the approved guidelines from the European directive 2010/63/UE and DL 4/03/2014 n 26 comma g. All experimental protocols requiring animal handling and the collection of samples were approved by the Institutional Animal Care and Use Committee of University of Perugia.

### Accession codes

The 16S rRNA gene amplicon sequence data supporting the conclusions of this article are available at the National Centre for Biotechnology Information Sequence Read Archive (SRA; http://www.ncbi.nlm.nih.gov/sra) bioproject number PRJNA397032, under the following SRA experiment accession numbers: SRR5895869 – SRR5895890.

## Results

### Animal performances and influence of polyphenol-enriched diets on rumen liquor FA and DMA

Three experimental groups of ewes were fed different diets based on alfalfa and extruded linseed as the α-LNA source. All diets were balanced and formulated according to the nutrient requirements of lactating ewes. A control diet (C) without any polyphenol supplementation and two treated diets including OOP obtained by a two-phase or a three-phase milling process (COOP2 and COOP3, respectively, Supplementary Table S1) were generated. During the trial, the administered concentrate was completely consumed by the animals regardless of the treatment (800 g/head/day). The average DMI was 2.26, 2.10 and 2.27 kg/head/day (SEM = ±0.19; P = 0.47) for the C, COOP2 and COOP3 groups, respectively.

The FA profile of RL from all groups was obtained. Significant differences (P < 0.05) between the control and treated groups were related to RA and α-LNA (Table 1). In particular, RA was significantly higher only in the RL of the COOP3 group, and α-LNA was significantly higher in ewes fed with COOP2 (45% increase respect to C group for both FAs). Moreover, the C16:1 *cis*7 content in the COOP3 group was lower than that in the other groups (Table 1).

**Table 1:** Effect of olive oil pomaces on FA^a^ production in rumen liquor.

FA^a^	Diet			P^b^
	g/100 g of FAs^a^ ± SEM^a^			
	C^a^	COOP2^a^	COOP3^a^	
12:0	0.131 ± 0.019	0.158 ± 0.0178	0.114 ± 0.019	0.261
13 *iso*	0.005 ± 0.001	0.005 ± 0.001	0.006 ± 0.001	0.583
13:0	0.028 ± 0.012	0.060 ± 0.011	0.032 ± 0.012	0.140
14 *iso*	0.084 ± 0.005	0.092 ± 0.005	0.089 ± 0.005	0.485
14:0	0.420 ± 0.081	0.641 ± 0.076	0.396 ± 0.081	0.074
15 *iso*	0.527 ± 0.042	0.525 ± 0.040	0.546 ± 0.042	0.930
15 *ante*	0.381 ± 0.022	0.388 ± 0.020	0.393 ± 0.022	0.928
15:0	0.462 ± 0.029	0.532 ± 0.027	0.460 ± 0.029	0.148
16 *iso*	0.211 ± 0.020	0.223 ± 0.019	0.226 ± 0.020	0.852
16:0	9.386 ± 0.739	8.034 ± 0.691	9.068 ± 0.739	0.388
17 *iso*	0.189 ± 0.014	0.189 ± 0.013	0.184 ± 0.014	0.963
16:1 *cis7*	0.191 ± 0.040a	0.237 ± 0.037a	0.090 ± 0.040b	0.043
16:1 *cis9*	0.032 ± 0.004	0.039 ± 0.004	0.032 ± 0.004	0.386
17 *ante*	0.264 ± 0.028	0.296 ± 0.026	0.297 ± 0.028	0.628
17:0	0.256 ± 0.016	0.245 ± 0.015	0.252 ± 0.016	0.885
18:0	25.494 ± 2.363	20.677 ± 2.210	25.502 ± 2.363	0.245
OA (18:1 *cis9*)^a^	4.598 ± 0.470	5.022 ± 0.440	5.183 ± 0.470	0.666
18:1 *cis11*	0.333 ± 0.087	0.188 ± 0.081	0.220 ± 0.087	0.464
18:1 *cis12*	0.157 ± 0.025	0.153 ± 0.023	0.144 ± 0.025	0.937
18:1 *cis13*	0.033 ± 0.004	0.030 ± 0.004	0.034 ± 0.004	0.763
18:1 *cis15*	0.167 ± 0.028	0.266 ± 0.026	0.211 ± 0.028	0.053
18:1 *trans5*	0.008 ± 0.001	0.008 ± 0.001	0.008 ± 0.001	0.989
18:1 *trans6-8*	0.095 ± 0.011	0.080 ± 0.011	0.086 ± 0.011	0.660
18:1 *trans9*	0.050 ± 0.005	0.041 ± 0.005	0.046 ± 0.005	0.514
18:1 *trans10*	0.075 ± 0.012	0.047 ± 0.012	0.052 ± 0.012	0.264
VA (18:1 *trans11*)1	0.382 ± 0.103	0.239 ± 0.097	0.167 ± 0.103	0.344
18:1 *trans12*	0.065 ± 0.008	0.058 ± 0.007	0.069 ± 0.008	0.602
18:1 *trans15*	0.066 ± 0.009	0.061 ± 0.008	0.071 ± 0.009	0.686
18:1 *trans16*	0.631 ± 0.72	0.464 ± 0.067	0.623 ± 0.072	0.183
18:2 *trans9 trans12*	0.106 ± 0.011	0.116 ± 0.010	0.113 ± 0.011	0.825
LA (18:2 *cis*9 *cis*12)^a^	0.582 ± 0.054	0.695 ± 0.051	0.712 ± 0.054	0.208
18:3 *trans9 trans12 trans15*	0.048 ± 0.016	0.021 ± 0.015	0.049 ± 0.016	0.349
20:0	0.320 ± 0.026	0.293 ± 0.025	0.302 ± 0.026	0.755
α-LNA (18:2 *cis*9 *cis*12 *cis*15)^a^	0.343 ± 0.029b	0.496 ± 0.023a	0.441 ± 0.029ab	0.004
RA (18:2 *cis*9 *trans*11)^a^	0.211 ± 0.021b	0.216 ± 0.020b	0.307 ± 0.021a	0.007
21:0	0.083 ± 0.011	0.084 ± 0.010	0.079 ± 0.011	0.944
22:0	0.188 ± 0.014	0.196 ± 0.013	0.194 ± 0.014	0.906
23:0	0.103 ± 0.005	0.094 ± 0.005	0.087 ± 0.005	0.162
24:0	0.203 ± 0.014	0.217 ± 0.013	0.217 ± 0.014	0.721

^a^Acronyms used in this table: FA (fatty acid), C (control diet), COOP2 (control diet added with olive oil pomace extracted with a two-phase procedure), COOP3 (control diet added with olive oil pomace extracted with a three-phase procedure), OA (oleic acid), VA (vaccenic acid), LA (linoleic acid), α-LNA (α linolenic acid), RA (rumenic acid) and SEM (Standard Error Mean).

^b^Probability of significant effect due to experimental diets; means within a row with different letters differ (P < 0.05).

Total DMA concentration did not vary among groups, and DMA16:0 was the most abundant DMA for all three diets (Table 2). Significant differences were found among the DMA profiles (3 out of 18 DMAs analyzed). DMA13:0 was higher in the RL of the C group than in the COOP2 and COOP3 groups. DMA18:0 reached the highest value when OOPs were included in the concentrates. DMA17:0 increased with OOP diet inclusion, with the highest value found in the COOP3 group (Table 2).

**Table 2:** Total DMA^a^ (mg/g dry matter ± SEM^a^) and DMA^a^ composition (g/100 g of total DMA^a^ ± SEM^a^) of rumen liquor.

DMA^a^	Diet			P^b^
	C^a^	COOP2^a^	COOP3^a^	
Total DMA	1.507 ± 0.118	1.775 ± 0.111	1.607 ± 0.119	0.267
DMA12:0	0.798 ± 0.218	1.244 ± 0.204	1.027 ± 0.218	0.348
DMAi13:0	2.434 ± 0.674	2.405 ± 0.630	1.931 ± 0.674	0.837
DMA13:0	1.149 ± 0.123a	0.729 ± 0.115b	0.587 ± 0.123b	0.012
DMAi14:0	6.039 ± 0.328	5.602 ± 0.307	5.202 ± 0.328	0.223
DMA14:0	8.044 ± 0.394	7.296 ± 0.369	6.785 ± 0.394	0.102
DMAi15:0	1.157 ± 0.204	1.391 ± 0.191	1.237 ± 0.204	0.697
DMAa15:0	5.639 ± 0.456	4.976 ± 0.435	4.712 ± 0.465	0.366
DMA15:0	6.168 ± 0.161	6.381 ± 0.151	6.010 ± 0.161	0.262
DMAi16:0	1.756 ± 0.233	1.655 ± 0.218	2.024 ± 0.233	0.507
DMA16:0	46.422 ± 1.754	46.625 ± 1.641	49.001 ± 1.754	0.518
DMA16:1	6.364 ± 0.763	6.209 ± 0.714	5.237 ± 0.763	0.534
DMAa17:0	2.617 ± 0.206	2.120 ± 0.192	2.419 ± 0.206	0.230
DMA17:0	0.482 ± 0.331c	1.564 ± 0.310b	2.989 ± 0.331a	<0.001
DMA18:0	2.481 ± 0.144b	3.127 ± 0.135a	3.051 ± 0.144a	0.009
DMA18:1t11	0.637 ± 0.070	0.693 ± 0.065	0.667 ± 0.070	0.844
DMA18:1c9	3.115 ± 0.380	3.901 ± 0.356	3.349 ± 0.380	0.317
DMA18:1c11	1.914 ± 0.244	1.836 ± 0.229	1.924 ± 0.244	0.958
DMA18:1c12	0.146 ± 0.074	0.334 ± 0.069	0.183 ± 0.074	0.166
DMA17:1	2.637 ± 0.540	1.910 ± 0.505	1.665 ± 0.540	0.430

^a^Acronyms used in this table: DMA (dimethyl acetal), C (control diet), COOP2 (control diet added with olive oil pomace extracted with a two-phase procedure), COOP3 (control diet added with olive oil pomace extracted with a three-phase procedure) and SEM (Standard Error Mean).

^b^Probability of significant effect due to experimental diets; means within a row with different letters differ (P < 0.05).

### Metataxonomy of rumen liquor (RL)

**Figure 1:**
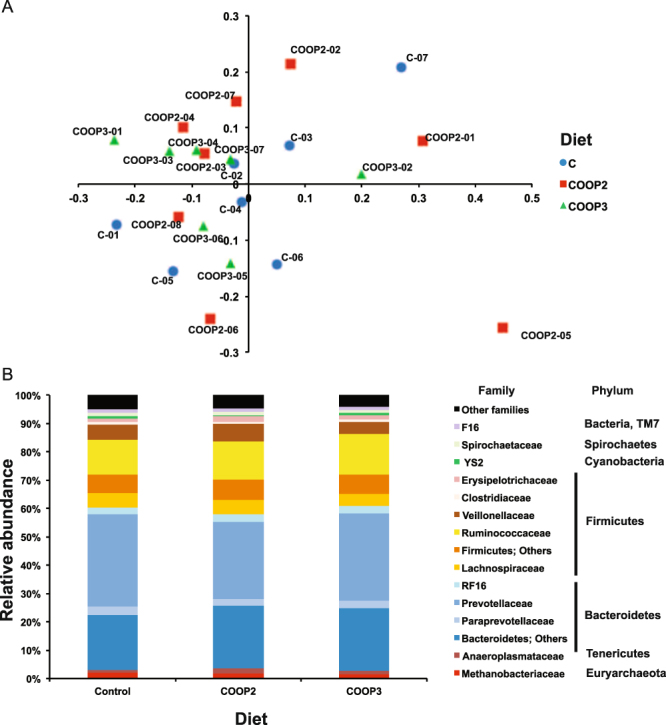
Rumen microbiota of ewes. Rumen microbiota are labeled respective to the ewe diet regimen (C = control diet; COOP2 = control diet added with olive oil pomace extracted with a two-phase procedure; COOP3 = control diet added with olive oil pomace extracted with a three-phase procedure). (**A**) ß-diversity, non-metric MDS plot. (**B**) Prokaryotic community composition of ewe RL at family level. Only families whose relative abundance was higher than 0.8% are shown.

The results of RL microbiota sequencing produced a total of 1,813 OTUs (based on 97% nucleotide sequence identity). In each sample, a similar number of OTUs was observed, with an average of 1,452 OTUs (ranging from 1,190 to 1,566). Rarefaction curves showed high sequencing coverage for all the samples (Supplementary Fig. S1). The α-diversity was calculated from the number of OTUs observed, and the Chao 1 value and Shannon diversity index did not differ significantly among the three groups (Supplementary Fig. S2). Addition of OOPs did not alter the overall microbiota composition, as indicated by the non-metric multidimensional scaling plot (nMDS, Fig. 1A), where all samples were evenly scattered. Sample COOP2-5 formed an out-group, and it was also one of the two specimens with the lowest number of OTUs observed (Supplementary Fig. S1). No significant differences related to diet were found with one-way ANOSIM and PERMANOVA (data not shown). The microbiota composition of the three groups was analyzed at different taxonomic levels. At the phylum level, the microbiota was dominated by *Bacteroidetes* and *Firmicutes* (approximately 56% and 32%, respectively) (Fig. 1B). Together, these two phyla accounted for 89 ± 0.7% of the total microbiota (Fig. 1B), but their relative abundance was highly variable (Supplementary Fig. S4), ranging from 0.9 to 2.9 (ratio *Bacteroidetes/Firmicutes*). *Prevotellaceae* was the most represented family (30%), followed by *Ruminococcaceae* (13.4%), *Veillonellaceae* (5.2%) and *Lachnospiraceae* (4.7%) (Fig. 1B).

**Figure 2:**
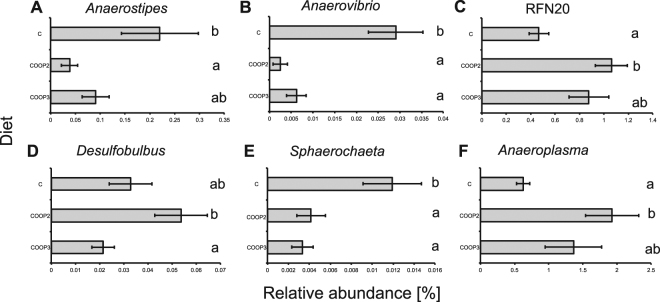
Effect of OOPs at genus level. Each bar is labeled respective to the ewe dietary regimen (C = control diet; COOP2 = control diet added with olive oil pomace extracted with a two-phase procedure; COOP3 = control diet added with olive oil pomace extracted with a three-phase procedure). Bacterial genera influenced by diet (ANOVA, p < 0,05): (**A**) *Anaerostipes*, (**B**) *Anaerovibrio*, (**C**) RFN20 genus, (**D**) *Desulfobulbus*, (**E**) *Sphaerochaeta* and (**F**) *Anaeroplasma*. Means sharing the same letter are not significantly different (post hoc Tukey’s HSD test).

*Anaerostipes, Anaerovibrio*, RFN20*, Anaeroplasma, Desulfobulbus* and *Sphaerochaeta* genera were influenced by OOP diet inclusion (Fig. 2). Excluding *Anaerostipes* and *Anaerovibrio*, the other four genera were the only representatives (in the present dataset) of their respective families and orders, so these differences were also reflected at higher taxonomic levels. Among the six different genera influenced by OOPs, the genus *Desulfobulbus* was the only *taxon* that had opposite behavior depending on the OOP used. The abundance of the genus *Desulfobulbus* was significantly higher in the COOP2 group than in the COOP3 group, but neither was significantly different from the C group. An increase in the relative abundance of RFN20 and *Anaeroplasma* was significant for the RL microbiota of ewes fed COOP2 (Fig. 2C and F). In particular, a three-fold increase in the *Anaeroplasma* genus was observed (Fig. 2F). This trend was opposite for *Anaerovibrio* and *Sphaerochaeta*, which resulted significantly lower (P < 0.01) in both OOP supplemented diets (Fig. 2B and E) respect to control diet. For the *Anaerovibrio* genus, a tenfold reduction was found in the RL microbiota of ewes fed COOP2, while in COOP3 was 80% less than that in the C group (Fig. 2B). A similar trend was observed for the *Sphaerochaeta* genus, which was the 65% and 72% less in COOP2 and COOP3 groups, respectively (Fig. 2E). The reduction in *Anaerostipes* abundance was significant only for the COOP2 group (P < 0.05, Fig. 2A).

### Pair-wise correlation

Pair-wise correlation showed that DMA13:0 was positively related to *Anaerostipes* (corr. coeff. +0.4661; P = 0.0287) and that DMA17:0 was positively related to *Sphaerochaeta* (corr. coeff. +0.4909; P = 0.0203), while DMA18:0 was positively correlated to RFN20 (corr. coeff. +0.5411; P = 0.0009) and to *Anaeroplasma* (corr. coeff. +0.4290; P = 0.0046) but negatively correlated to *Anaerovibrio* (corr. coeff. −0.5836; P = 0.0043).

*Desulfobulbus* was positively related to DMAi13:0 (corr. coeff. +0.4702; P = 0.02723). Considering the FAs involved in BH processes, *Anaerovibrio* was negatively correlated with α-LNA (corr. coeff. −0.6096; P = 0.0025) and LA (corr. coeff. −0.4795; P = 0.0239), while RFN20 genus was positively related to α-LNA (corr. coeff. +0.5789; P = 0.0047) only. RA showed a negative correlation with *Veillonellaceae* (corr. coeff. −0.4363; P = 0.0423) and a positive correlation with *Coriobacteriaceae* (corr. coeff. +0.5425; P = 0.0090).

### Milk composition and FA profile

Dietary treatments did not significantly affect milk yield and composition (Table 3). The FA composition of milk from ewes fed COOP2 was significantly different from that in the C group (Table 4). In contrast, the effect of the COOP3 diet was intermediate between the COOP2 and C diets (Table 4). In particular, the content of several short- and medium-chain fatty acids (SMCFAs, 6:0, 8:0, 10:0, 10:1 cis9, 12:0 and 14:0) was lower in COOP2 milk samples than in the other samples. Moreover milk from ewes fed COOP2 was higher in unsaturated long-chain fatty acids (ULCFAs), such as oleic acid (18:1 *cis*9, OA) and α-LNA.

**Table 3:** Effect of olive oil pomaces on milk yield and composition.

	Diet			SEM^a^	P^b^
	C^a^	COOP2^a^	COOP3^a^		
Milk yield (g/days)	575.27	603.58	617.21	80.92	0.72
FCM^a^ (g/days)	698.06	699.31	744.56	75.41	0.69
Milk fat (g/100 g)	8.65	8.19	8.62	0.28	0.52
Milk protein (g/100 g)	6.52	6.07	6.38	0.17	0.89
Milk lactose (g/100 g)	4.41	4.54	4.38	0.07	0.53
Milk urea (mg/dL)	49.00	37.97	46.85	4.24	0.54
Linear Score^c^	3.66	2.95	3.93	0.44	0.65

^a^Acronyms used in this table: C (control diet), COOP2 (control diet added with olive oil pomace extracted with a two-phase procedure), COOP3 (control diet added with olive oil pomace extracted with a three-phase procedure), SEM (Standard Error Mean) and FCM (Fat-Corrected Milk).

^b^Probability of significant effect due to experimental diets; means within a row with different letters differ (P < 0.05).

^c^Linear score: log2 (Somatic cell count/12,500).

**Table 4:** Effect of olive oil pomaces on FAs^a^ production in milk.

FA^a^	Diet			SEM^a^	P^b^
	g/100 g of FAs^a^				
	C^a^	COOP2^a^	COOP3^a^		
4:0	2.65	2.81	2.67	0.08	0.28
6:0	1.73a	1.33b	1.63ab	0.10	0.02
8:0	1.49a	0.98b	1.33ab	0.11	0.01
10:0	4.22a	2.62b	3.69ab	0.35	0.01
10:1 *cis*9	0.14a	0.08b	0.13ab	0.02	0.03
11:0	0.04	0.03	0.03	<0.01	0.08
12:0	2.42a	1.67b	2.15ab	0.17	0.01
13:0 *iso*	0.01	0.02	0.01	<0.01	0.18
13:0 *anteiso*	0.01	0.01	0.01	<0.01	0.06
12:1 *cis*11	0.02	0.02	0.02	<0.01	0.77
13:0	0.04	0.04	0.04	<0.01	0.60
14:0 *iso*	0.07	0.08	0.07	<0.01	0.15
14:0	6.95a	5.64b	6.61ab	0.33	0.02
15:0 *iso*	0.16	0.18	0.15	0.01	0.10
15:0 *anteiso*	0.27	0.29	0.26	0.01	0.35
14:1 *cis*9	0.11	0.08	0.12	0.01	0.08
15:0	0.69	0.77	0.69	0.03	0.05
16:0 *iso*	0.15	0.15	0.14	0.01	0.72
16:0	16.72	15.62	16.86	0.42	0.09
16:1 *trans*9	0.28	0.34	0.28	0.02	0.14
17:0 *iso*	0.26	0.28	0.26	0.01	0.35
16:1 *cis*7	0.24b	0.28a	0.24b	0.01	0.02
16:1 *cis*9	0.54	0.46	0.57	0.03	0.06
17:0 *anteiso*	0.23	0.25	0.23	0.01	0.20
17:0	0.42	0.49	0.43	0.02	0.07
17:1 *cis*9	0.12	0.13	0.12	0.01	0.23
18:0	9.22	9.72	9.72	0.55	0.75
18:1*trans6-8*	0.91	1.01	0.89	0.04	0.13
18:1 *trans*9	0.69	0.75	0.73	0.05	0.66
18:1 *trans*10	0.90	0.96	0.95	0.08	0.84
VA (18:1 *trans*11)^a^	4.24	4.64	4.29	0.33	0.63
18:1 *trans*12	0.85	0.85	0.84	0.02	0.96
OA (18:1 *cis*9)^a^	19.53b	22.26a	21.06a	0.69	0.03
18:1 *trans*15	0.45b	0.52a	0.48ab	0.02	0.02
18:1 *cis*11	0.43	0.41	0.45	0.02	0.29
18:1 *cis12*	0.34	0.31	0.31	0.02	0.33
18:1 *trans16*	0.51	0.54	0.53	0.02	0.57
18:1 *cis14*	0.07	0.08	0.08	0.01	0.27
LA (18:2 *cis*9 *cis*12)^a^	1.86	1.98	1.95	0.11	0.68
α-LNA (18:2 *cis*9 *cis*12 *cis*15)^a^	1.90b	2.38a	1.90b	0.13	0.02
RA (18:2 *cis*9 *trans*11)^a^	2.03	2.25	2.19	0.14	0.53
18:3 *cis*9 *trans*11 *cis*15	0.05b	0.06a	0.04b	<0.01	0.02
20:0	0.13b	0.17a	0.14b	0.01	0.01
20:3n6	0.02	0.02	0.02	<0.01	0.63
20:3n3	0.03	0.03	0.03	<0.01	0.20
20:4n6	0.10	0.11	0.11	0.01	0.32
20:5n3	0.05	0.06	0.06	<0.01	0.23
22:5n3	0.10	0.12	0.10	0.01	0.13
22:6n3	0.04	0.04	0.04	<0.01	0.51

^a^Acronyms used in this table: FA (fatty acid), C (control diet), COOP2 (control diet added with olive oil pomace extracted with a two-phase procedure), COOP3 (control diet added with olive oil pomace extracted with a three-phase procedure), OA (oleic acid), VA (vaccenic acid), LA (linoleic acid), α-LNA (α linolenic acid), RA (rumenic acid) and SEM (Standard Error Mean).

^b^Probability of significant effect due to experimental diets; means within a row with different letters differ (P < 0.05).

## Discussion

RL microorganisms are highly sensitive to dietary composition and, in particular, to supplements with antimicrobial activity, such as polyphenols^38^. Much of our knowledge related to rumen metabolism of feeds and additives has been gained by *in vitro* studies^13^. Thus, it is necessary to perform *in vivo* trials for better understanding the effects of diet quality. Pomace is a by-product of the olive oil extraction process and is rich in polyphenols, the amount of which is variable depending on the production technique used (two-phase *vs* three-phase method). The addition of polyphenols to animal diets may alter rumen microorganism activities^4,13,39^. The lipid content of milk and meat is influenced by rumen metabolism. Thus, modulation of RL microbiota to increase the amount of nutraceutical PUFAs may be exploitable to ameliorate food production.

In this trial results related to DMI did not show significant differences among experimental groups. Therefore, the effects of dietary supplementation with OOPs on RL and milk composition were due to the different chemical profiles of the experimental diets. OOP addition led to an increase in PUFAs. In particular, a gain of α-LNA was obtained with OOP2, while the RA concentration was enhanced with OOP3. The different changes observed in FAs among ewe groups may be due to the different contents of polyphenols into the two extracts used as supplements in the treated diets. COOP2 showed a higher content of verbascoside, 3,4 DHPEA-EDA, and rutin than COOP3, as a consequence of the different extraction processes. Verbascoside is a molecule with antioxidant, anti-inflammatory and antimicrobial activities^40^, suggesting that it may play a role in the modulation of rumen microbiota.

Since the 1980s, chemotaxonomic techniques have been considered of important value to identify and classify bacterial strains in rumen microbial ecosystems^41^. Several authors have found that DMAs are associated with specific bacterial *taxa*^41–44^, indicating that plasmalogen lipid profiles may be considered a tool for microbial community characterization. These molecules are present in bacterial membrane, especially of anaerobic species. However, their function is not completely known. DMAs play a key role in the regulation of membrane fluidity, and their profile changes when environmental conditions vary. The DMA profile reflects the FAME composition, and a characteristic DMA profile could be associated with a specific microbial strain^41–44^. In this study, DMA 16:0 was the most abundant in all groups, which was in agreement with previous results of Alves *et al*.^22^. Significant variations were observed for DMA13:0, DMA17:0 and DMA18:0, even if the total DMA concentration did not vary among groups.

Metataxonomic analysis of RL microbiota showed that overall composition was unaffected by OOP addition. Although unrelated to diet composition high variability was observed, especially in the relative abundance of *Bacteroidetes* and *Firmicutes*. Moreover, in this trial, a low abundance of the *Lachnospiraceae* family (<5%) was found, and it has been reported to be generally higher (>10%) in ewe rumen microbiota^45^. The low abundance of *Lachnospiraceae* may be linked to the addition of a high content of concentrate (rich in starch and fat, and poor in fiber and polysaccharide xylan, which are the main substrates for cellulolytic bacteria growth) in all the ewe dietary regimens. However, the method used for rumen fluid collection may also affect solid-associated bacteria content because the esophageal pump may be selective in feed particle extraction, although it is a general veterinary practice. The *Lachnospiraceae* family is mainly represented by the genus *Butyrivibrio*, accounting for 1% of the total microbiota, and it has been known for its role in BH since the 1960s^46^. An increase in α-LNA and RA contents was observed upon OOP2 or OOP3 addition to the diet, which may be due to a decrease in BH or to lower availability of substrates for BH. Although the *Butyrivibrio* relative presence did not vary among groups, other microorganisms are known to be involved in the BH process (i.e., *Megasphaera elsdenii* and *Propionibacterium acnes*) and have been characterized in recent decades^1^. Furthermore, a putative new role has recently been assigned to several known microorganisms, which were considered until now to be involved in other processes^47,48^.

In this study, variations linked to diet were observed for *Anaerostipes, Anaerovibrio*, RFN20*, Anaeroplasma, Desulfobulbus* and *Sphaerochaeta*. Within *Firmicutes*, the genus *Anaerovibrio* was less represented in animals fed with OOP. For *Anaerostipes*, the decrease was significant only for COOP2. *Anaerostipes* is a butyrate-producing bacterium whose activity is strongly linked to fermentation of dietary carbohydrates, and its lower relative abundance in RL from ewes fed COOP2 may be related to the lower quality of fiber contained in this diet as lignin is indigestible^49^. The opposite behavior was observed for bacteria belonging to RFN20 genus, which was significantly higher in the COOP2 group, but the role of this *taxon* within the rumen microbial community remains unclear^50^. Similarly, *Anaeroplasma* was higher in the COOP2 diet. These microorganisms are anaerobic mycoplasmas, which in some cases have bacteriolytic capabilities affecting nutrient cycling and protein turnover. Indeed, their activity may reduce Gram-negative bacteria, thus interfering with rumen processes^51^. Mycoplasmas may also parasite ruminal fungi and protozoa modifying their activity^51^. Pair-wise analysis showed that a variation of DMA13:0 may be related to a variation of *Anaerostipes*, and that changes in DMA17:0 may be related to changes in *Sphaerochaeta*, whereas DMA18:0 variations may be related to a variation of *Anaeroplasma*, RFN20 and *Anaerovibrio*.

Increase in PUFAs may be linked to the lower abundance of the *Anaerovibrio* in the RL microbiota. This relation was confirmed by the pair-wise correlation, which showed a significant and negative correlation between α-LNA and *Anaerovibrio* content (corr. coeff. = −0.6096, P = 0.0026). *Anaerovibrio lipolyticus* is the only species described within this genus, and it is a key player in the lipolysis process^52^. Lipolysis is a fundamental requirement for the next step of lipid metabolism in RL, bacterial membrane structure formation, cell replication and PUFA-BH. *A. lipolyticus* growth is enhanced with diets having a high content of concentrate respect to the diet based on hay^53^. Hence, according to the high level of concentrate used in this trial, an increase in *A. lipolyticus* was expected in all ewe groups. In contrast, the decrease of *A. lipolyticus* in the COOP2 and COOP3 groups respect to the C group, showed the negative action of OOP on *A. lipolyticus* growth. This bacterium uses glycerol as a nutrient^54,55^. Thus, it could be hypothesized that the presence of polyphenols in the diets may have complexed lipase enzymes, avoiding the triglyceride hydrolysis^56,57^ and ultimately resulting in less free glycerol available to *A. lipolyticus* for its growth^58,59^. Reduction of *A. lipolyticus* led to a decrease in lipolytic activity, and hence, to a low availability of PUFAs for BH, which agreed with the increase of α-LNA and RA. It has been reported that polyphenols, such as those from chestnut or quebracho, interfere with the last step of the BH process, inhibiting the activity of microorganism, such as *B. proteoclasticum*^3,39^. This study showed that polyphenols from OOP act in a different manner by affecting *A. lipolyticus* abundance and consequently lipolysis, which is the step before BH. The lower abundance of *A. lipolyticus* may explain the differences observed for PUFA concentrations in the RL of animals fed with OOP diets. Nevertheless, considering the putative role of *Butyrivibrio* group in the BH, it is possible that within the *Butyrivibrio* genus, the relative abundance of different *Butyrivibrio* species (i.e., *B. fibrisolvens*) may affect the BH process.

Dietary treatment did not significantly affect milk yield and composition. Inconsistent results have been reported in previous studies, and these differences are probably due to the inclusion levels of OOP and/or to their associative effects with specific diets^12,60,61^. Regarding milk urea content, the results suggested that olive phenols do not interact with the dietary protein metabolism unlike to other phenolic substances, such as tannins^62^. The lower content of several SMCFAs in milk fat from ewes fed COOP2 should be related to a lowering of mammary gland *de novo* fat synthesis^63^. Considering long-chain fatty acids, OA content was higher in milk fat from ewes fed OOP diets than with control, although the OA supply was similar across diets. OA is largely generated by mammary Δ^9^-desaturation of 18:0^64^. Further studies are required to better understand the role of OOPs on mammary gland metabolism. An increased content in α-LNA was found only in milk fat from ewes fed COOP2, as observed in the RL of the same animals. In contrast, no significant differences in RA concentrations in milk fat were observed, which may have been due to mammary gland activity for the Δ^9^-desaturation of VA^64^.

## Conclusions

The use of different types of OOP in dairy ewe diets did not negatively affect milk yield or composition. COOP2 and COOP3 diets led to an enrichment of the milk fat with α-LNA and OA. The changes in microbiota profile due to OOPs are limited and do not alter rumen functionality, preserving animal welfare. The data of this trial highlighted that *A. lipolyticus* is particularly sensitive to OOPs. Hence, depending on the types of polyphenol added to diet, it might be possible to modulate rumen metabolism at different levels as they affect relative abundance of different microorganisms related to BH. In conclusion, this study suggested that OOPs may be used in ruminant feeding because they induce a decrease in lipolysis, favoring the accumulation of healthy FAs in milk.

## Electronic supplementary material

Supplementary information

Supplementary dataset
