# Supplementary material for: Effect of different types of olive oil pomace dietary supplementation on the rumen microbial community profile in Comisana ewes
Source: Sci Rep. 2018 May 31;8:8455. doi: 10.1038/s41598-018-26713-w (PMC5981327; doi:10.1038/s41598-018-26713-w)
Supplement: Supplementary file 1 — Supplementary information [file 41598_2018_26713_MOESM1_ESM.docx]

**Effect of different types of olive oil pomace dietary supplementation on the rumen microbial community profile in Comisana ewes**.

Federica Mannelli^1+^, Alice Cappucci^2+^, Francesco Pini^1+^, Roberta Pastorelli^5^, Francesca Decorosi^1^, Luciana Giovannetti^1^, Marcello Mele^2^, Sara Minieri^4^, Giuseppe Conte^2^, Mariano Pauselli^4^, Stefano Rapaccini^1^, Carlo Viti^1*^ and Arianna Buccioni^1^.

**List of supplementary information**

**Supplementary methods**

**Figure S1.** Sample-based rarefaction curves representing the number of observed OTUs at different sequencing depths (each point is the average of 10 iterations). RL microbiota are labeled respect to the ewe diet regimen (C = control diet; COOP2 = control diet added with OOP extracted with a two-phase procedure; COOP3 = control diet added with OOP extracted with a three-phase procedure).

**Figure S2.** Box-plots of bacterial α-Diversity based on: A) Observed OTUs, B) Chao1 value and C) Shannon index. Each box is labeled respect to the ewe diet regimen (C = control diet; COOP2 = control diet added with OOP extracted with a two-phase procedure; COOP3 = control diet added with OOP extracted with a three-phase procedure).

**Figure S3.** Prokaryotic microbiota composition (expressed as relative abundance) for each sample at phylum level. Bars are labeled respect to the ewe diet regimen (C = control diet; COOP2 = control diet added with OOP extracted with a two-phase procedure; COOP3 = control diet added with OOP extracted with a three-phase procedure).

**Table S1.** Ingredients (g/kg of DM) of the experimental diets used.

**Table S2.** Chemical composition (g/kg DM) of the experimental diets used in this trial.

**Table S3.** Primer and barcode sequences used in this work.

**Table S4.** QIIME taxa table of rumen microbiota composition for each sample at phylum level.

**Table S5.** QIIME taxa table of rumen microbiota composition for each sample at class level.

**Table S6.** QIIME taxa table of rumen microbiota composition for each sample at order level.

**Table S7.** QIIME taxa table of rumen microbiota composition for each sample at family level.

**Table S8.** QIIME taxa table of rumen microbiota composition for each sample at genus level.

**Supplementary methods**

**Feed sampling and analysis**

Offered hay and orts were daily weighed and weekly sampled to be analyzed. Samples were stored at -80°C, freeze dried (Minifast D2000 Edwards) and were grounded by mill Cyclotec 1093 (PBI International, Italy), using a mesh size of 1 mm. Crude protein (CP), ether extract (EE) and ash were determined according to the AOAC methods^1^. Neutral detergent fibre (NDF), acid detergent fibre (ADF) and lignin were determined according to Van Soest *et al*.^2^, using heat stable amylase and sodium sulphite. Results were expressed inclusive of residual ash. Fatty acid (FA) of feed samples were extracted according to Folch *et al*.^3^ and methylated according to Christie^4^, using valeric acid (5:0) and nonadecanoic acid (19:0) methyl esters (Sigma Chemical Co., St. Louis, MO) as internal standard. Gas-chromatography procedures and methods applied to identify and quantify feed FA (Supplementary Table S2), were the same described for rumen FA analysis.

**Fatty acids Gas Chromatograph programming**

Individual FAME were identified by comparison of the relative retention times of FAME peaks from samples, with those of the standard mixture 52 Component FAME Mix (Nu-Chek Prep Inc., Elysian, MN), 77 individual FAME standards (Larodan Fine Chemicals, Malmo, Sweden) and individual 18:1 *trans*9 and 18:1 *trans*11 (Sigma-Aldrich, St. Louis, MO), 18:2 *cis*9, *trans*11 (Matreya Inc., Pleasant Gap, PA), CLA mix standard (Sigma-Aldrich, St. Louis, MO), and published isomeric profiles^5-7^. The 18:1 isomer elution sequence was performed according to Kramer *et al*.^6^. Moreover, a standard mix of α-LNA isomers, LA isomers (Supelco, Bellefonte, PA) and published isomeric profiles^8^ were used to identify the isomers of interest. Two bacterial acid methyl ester mixes (Supelco, Bellefonte, PA) and individual standard for methyl ester of *iso* 14:0, *anteiso* 14:0, *iso* 15:0, and *anteiso* 17:0 (Larodan, Malmo, Sweden) were used to identify the branched FA profile. Inter- and intra-assay coefficients of variation were calculated by using a reference standard butter (CRM 164, Community Bureau of Reference, Brussels, Belgium), and the detection threshold of FA was 0.01 g / 100 g of FA^9^.


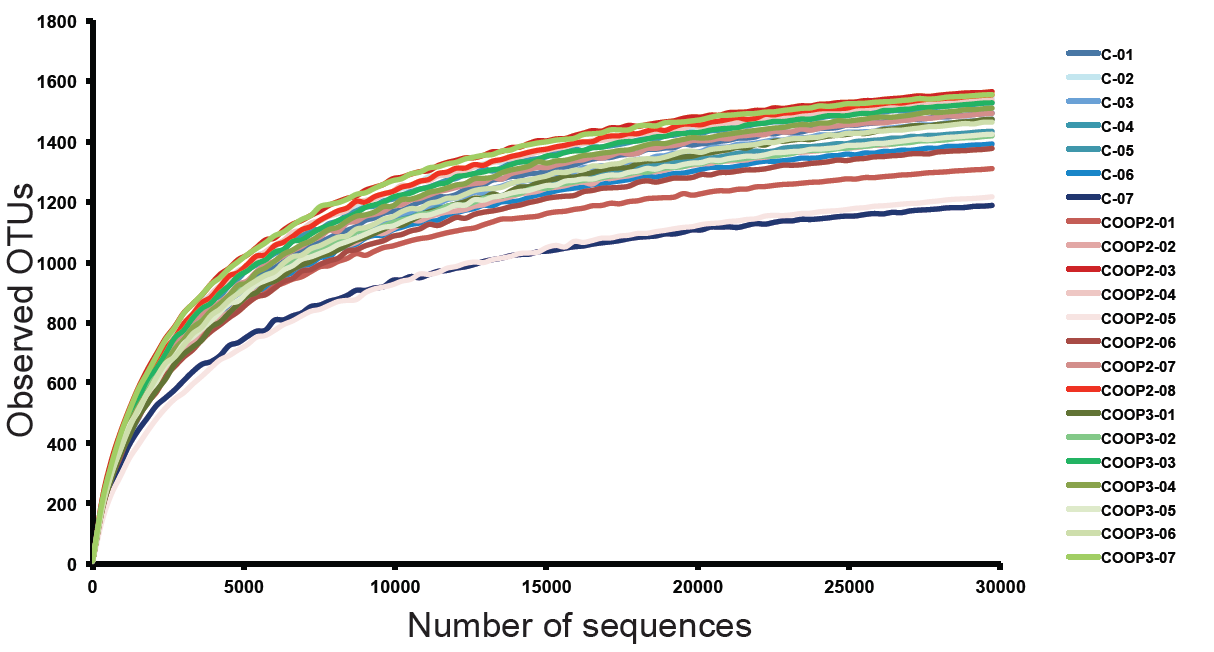


**Figure S1.** Sample-based rarefaction curves representing the number of observed OTUs at different sequencing depths (each point is the average of 10 iterations). RL microbiota are labeled respect to the ewe diet regimen (C = control diet; COOP2 = control diet added with OOP extracted with a two-phase procedure; COOP3 = control diet added with OOP extracted with a three-phase procedure).


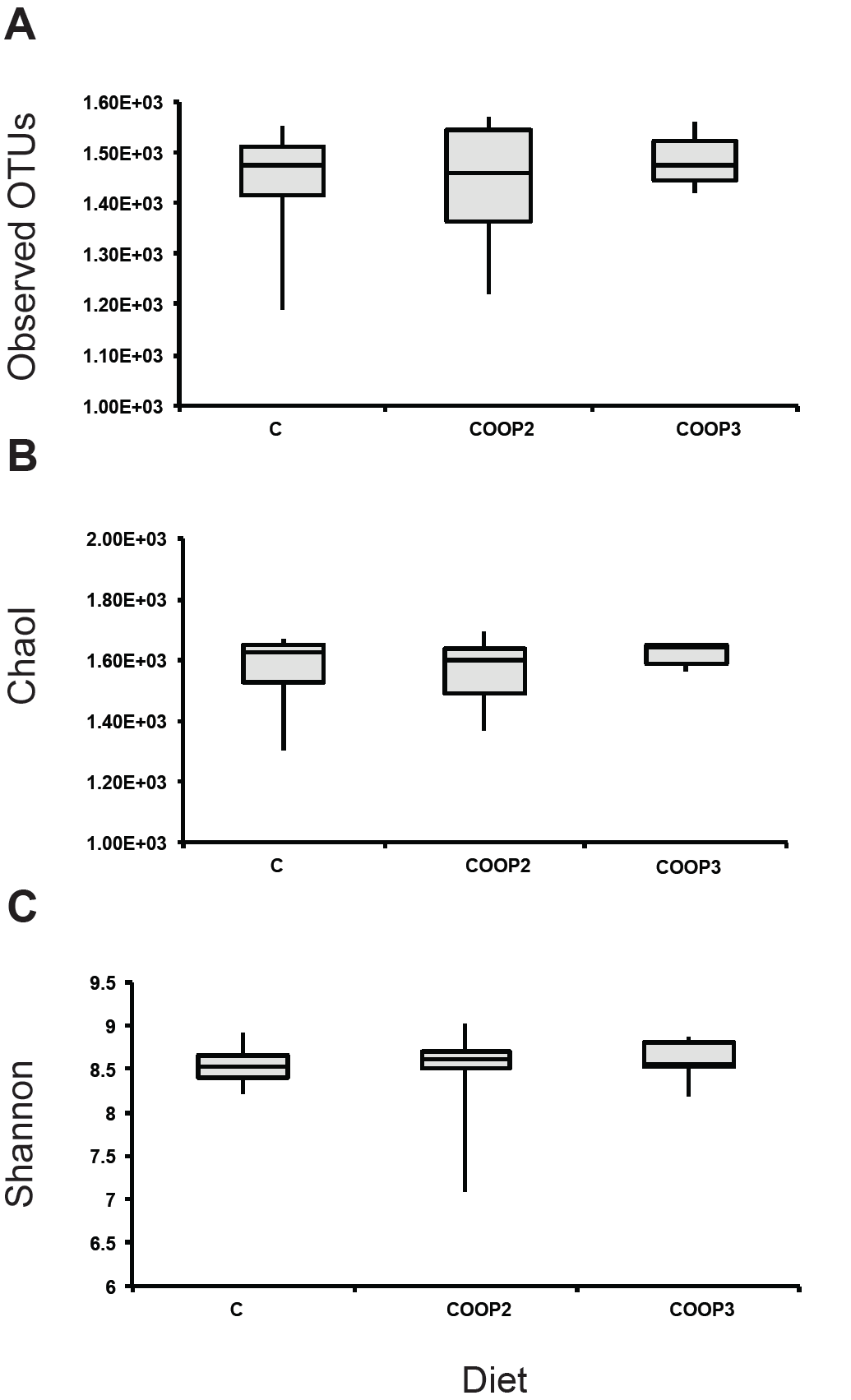


**Figure S2.** Box-plots of bacterial α-Diversity based on: A) Observed OTUs, B) Chao 1 value and C) Shannon index. Each box is labeled respect to the ewe diet regimen (C = control diet; COOP2 = control diet added with OOP extracted with a two-phase procedure; COOP3 = control diet added with OOP extracted with a three-phase procedure).


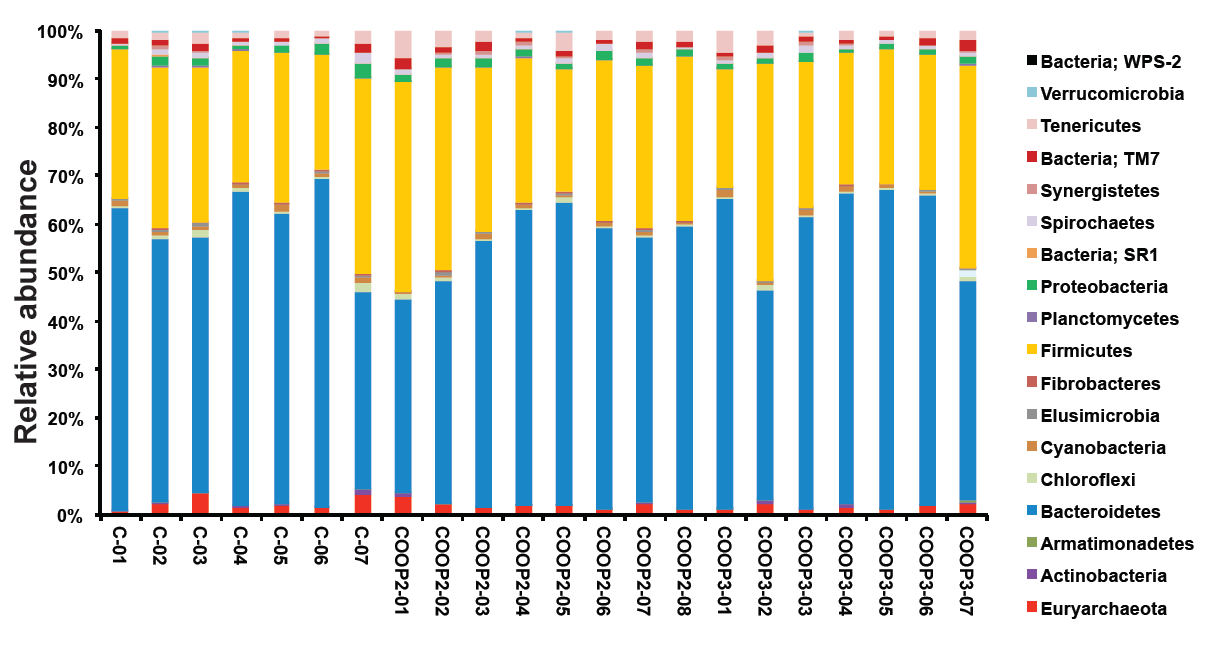


**Figure S3.** Prokaryotic microbiota composition (expressed as relative abundance) for each sample at phylum level. Bars are labeled respect to the ewe diet regimen (C = control diet; COOP2 = control diet added with OOP extracted with a two-phase procedure; COOP3 = control diet added with OOP extracted with a three-phase procedure).

**Table S1.** Ingredients (g/kg of DM^1^) of the experimental diets used.

| **Ingredients (g / kg of DM^1^)** | **Experimental concentrates** | | |
| --- | --- | --- | --- |
|  | **C^1^** | **COOP2^1^** | **COOP3^1^** |
| Wheat bran | 201.9 | 20.2 | 201.9 |
| Corn | 102.3 | 30.7 | 102.1 |
| Broad bean | 30.6 | 30.7 | 20.4 |
| Sunflower meal | 10.2 | 25.6 | 81.7 |
| Corn gluten | 10.2 | 20.4 | 10.2 |
| Dehydrated alfalfa | 201.9 | 272.9 | - |
| Barley | 126.2 | 50.5 | 80.8 |
| Molasses | 50.1 | 50.2 | 50.1 |
| Stoned olive pomace | - | 272.9 | 227.1 |
| Extruded linseed | 199.6 | 199.8 | 199.6 |
| Olive oil | 40.8 | - | - |
| CaCO_3_ | 10.4 | 10.4 | 10.4 |
| Sodium bicarbonate | 5.2 | 5.2 | 5.2 |
| Di-calcium phosphate | 5.2 | 5.2 | 5.2 |
| Sodium chloride | 5.2 | 5.2 | 5.2 |

^1^ DM = Dry matter; C = control diet; COOP2 = control diet added with OOP extracted with a two-phase procedure; COOP3 = control diet added with OOP extracted.

**Table S2.** Chemical composition (g/kg of DM^1^) of the experimental diets used in this trial.

|  | **Alfalfa hay** | **Rolled barley** | **Experimental concentrates** | | |  |
| --- | --- | --- | --- | --- | --- | --- |
|  |  |  | **C^1^** | **COOP2^1^** | **COOP3^1^** | |
| ***Chemical composition (g / kg of DM^1^)*** | | | | | |  |
| CP | 139.9 | 118.6 | 148.9 | 153.8 | 149.1 |  |
| EE | 11.7 | 34.4 | 129.6 | 121.4 | 127.4 |  |
| NDF | 499.8 | 269.3 | 290 | 318.4 | 300.5 |  |
| ADF | 349.7 | 102.5 | 149 | 195.2 | 151.7 |  |
| ADL | 110.6 | 24.4 | 37.7 | 78.8 | 97.8 |  |
| Ash | 77.4 | 26.8 | 68.1 | 91.8 | 64.7 |  |
| ***FA (% of total FAME)*** | | | | | |  |
| C16:0 | 24.5 | 23.6 | 9.0 | 7.8 | 8.6 |  |
| C16:1 | 2.8 | 1.2 | 0.4 | 0.2 | 0.3 |  |
| C18:0 | 5.1 | 1.8 | 2.7 | 2.8 | 2.6 |  |
| C18:1 cis-9 | 5.4 | 18.8 | 34.1 | 36.6 | 33.9 |  |
| C18:2 cis-9,cis-12 | 21.0 | 47.4 | 19.4 | 16.4 | 19.9 |  |
| C18:3 n-3 | 39.6 | 4.4 | 31.5 | 33.6 | 31.8 |  |
| SFA | 31.2 | 27.5 | 12.0 | 10.9 | 11.4 |  |
| MUFA | 8.2 | 20.0 | 36.3 | 38.0 | 36.0 |  |
| PUFA | 60.6 | 51.8 | 51.6 | 51.0 | 52.2 |  |
| **Polyphenol profile (g/kg of DM^1^)** | | | | | |  |
| 3,4-DHPEA | - | - | - | 1.2 | 1.0 |  |
| p-PEA | - | - | - | 0.3 | 0.2 |  |
| Verbascoside | - | - | - | 1.1 | 0.3 |  |
| 3,4-DHPEA-EDA | - | - | - | 2.1 | 1.0 |  |
| Rutin | - | - | - | 0.2 | 0.1 |  |
| Total polyphenols | - | - | - | 4.9 | 2.7 |  |

^1^ DM = Dry matter; C = control diet; COOP2 = control diet added with OOP extracted with a bi-phasic procedure; COOP3 = control diet added with OOP extracted with a three-phasic procedure; FA = Fatty acid; FAME = Fatty acid methyl ester.

**Table S3.** Primer and barcode sequences used in this work.

^1^C = control diet; COOP2 = control diet added with OOP extracted with a bi-phasic procedure; COOP3 = control diet added with OOP extracted with a three-phasic procedure.

| Sample ID | BarcodeSequence | LinkerPrimerSequence | Treatment | Reverseprimer | Description |
| --- | --- | --- | --- | --- | --- |
| CD-01 | AAAAAAAAAAAACCCC | TCCTACGGGAGGCAGCAGT | C^1^ | TCCTACGGGAGGCAGCAGT | MicrobIT10 |
| CD-02 | AAAAAAAAAAAAGGGG | TCCTACGGGAGGCAGCAGT | C | TCCTACGGGAGGCAGCAGT | MicrobIT16 |
| PSD-01 | AAAAAAAAAAAAAACC | TCCTACGGGAGGCAGCAGT | COOP3^1^ | TCCTACGGGAGGCAGCAGT | MicrobIT6 |
| CD-03 | TAAAAAAAAAAAAACC | TCCTACGGGAGGCAGCAGT | C | TCCTACGGGAGGCAGCAGT | MicrobIT22 |
| CD-04 | AAAAAAAAAAAAAAAA | TCCTACGGGAGGCAGCAGT | C | TCCTACGGGAGGCAGCAGT | MicrobIT21 |
| CSD-01 | AAAAAAAAAAAAAATT | TCCTACGGGAGGCAGCAGT | COOP2^1^ | TCCTACGGGAGGCAGCAGT | MicrobIT3 |
| CD-05 | AAAAAAAAAAAAAAGG | TCCTACGGGAGGCAGCAGT | C | TCCTACGGGAGGCAGCAGT | MicrobIT20 |
| CD-06 | TTAAAAAAAAAAAAAA | TCCTACGGGAGGCAGCAGT | C | TCCTACGGGAGGCAGCAGT | MicrobIT5 |
| PSD-02 | GGCCAAAAAAAAAAAA | TCCTACGGGAGGCAGCAGT | COOP3 | TCCTACGGGAGGCAGCAGT | MicrobIT1 |
| CSD-02 | AAAAAAAAAAAATTAA | TCCTACGGGAGGCAGCAGT | COOP2 | TCCTACGGGAGGCAGCAGT | MicrobIT13 |
| CD-07 | AAAAAAAAAAAAGGTT | TCCTACGGGAGGCAGCAGT | C | TCCTACGGGAGGCAGCAGT | MicrobIT19 |
| PSD-03 | AAAAAAAAAAAAGGCC | TCCTACGGGAGGCAGCAGT | COOP3 | TCCTACGGGAGGCAGCAGT | MicrobIT18 |
| PSD-04 | AAAAAAAAAAAACCAA | TCCTACGGGAGGCAGCAGT | COOP3 | TCCTACGGGAGGCAGCAGT | MicrobIT9 |
| CSD-03 | AAAAAAAAAAAATTCC | TCCTACGGGAGGCAGCAGT | COOP2 | TCCTACGGGAGGCAGCAGT | MicrobIT14 |
| PSD-05 | AAAAAAAAAAAATTGG | TCCTACGGGAGGCAGCAGT | COOP3 | TCCTACGGGAGGCAGCAGT | MicrobIT12 |
| CSD-04 | AAAAAAAAAAAACCTT | TCCTACGGGAGGCAGCAGT | COOP2 | TCCTACGGGAGGCAGCAGT | MicrobIT11 |
| CSD-05 | TTCCAAAAAAAAAATT | TCCTACGGGAGGCAGCAGT | COOP2 | TCCTACGGGAGGCAGCAGT | MicrobIT7 |
| PSD-06 | GGAAAAAAAAAAAACC | TCCTACGGGAGGCAGCAGT | COOP3 | TCCTACGGGAGGCAGCAGT | MicrobIT2 |
| CSD-06 | TTAAAAAAAAAAAAGG | TCCTACGGGAGGCAGCAGT | COOP2 | TCCTACGGGAGGCAGCAGT | MicrobIT4 |
| CSD-07 | AAAAAAAAAAAATTTT | TCCTACGGGAGGCAGCAGT | COOP2 | TCCTACGGGAGGCAGCAGT | MicrobIT15 |
| CSD-08 | AAAAAAAAAAAACCGG | TCCTACGGGAGGCAGCAGT | COOP2 | TCCTACGGGAGGCAGCAGT | MicrobIT8 |
| PSD-07 | AAAAAAAAAAAAGGAA | TCCTACGGGAGGCAGCAGT | COOP3 | TCCTACGGGAGGCAGCAGT | MicrobIT17 |

**References**

1 Cunniff, P. & Association of Official Analytical Chemists. *Official methods of analysis of AOAC international*. (Association of Official Analytical Chemists, 1995).

2 Van Soest, P. J., Robertson, J. B. & Lewis, B. A. Methods for dietary fiber, neutral detergent fiber, and nonstarch polysaccharides in relation to animal nutrition. *Journal of Dairy Science* **74**, 3583-3597, doi:10.3168/jds.S0022-0302(91)78551-2 (1991).

3 Folch, J., Lees, M. & Sloane Stanley, G. H. A simple method for the isolation and purification of total lipides from animal tissues. *The Journal of Biological Chemistry* **226**, 497-509 (1957).

4 Christie, W. W. A simple procedure for rapid transmethylation of glycerolipids and cholesteryl esters. *Journal of Lipid Research* **23**, 1072-1075 (1982).

5 Kramer, J. K. *et al.* Evaluating acid and base catalysts in the methylation of milk and rumen fatty acids with special emphasis on conjugated dienes and total trans fatty acids. *Lipids* **32**, 1219-1228 (1997).

6 Kramer, J. K. *et al.* Analysis of conjugated linoleic acid and trans 18:1 isomers in synthetic and animal products. *The American Journal of Clinical Nutrition* **79**, 1137S-1145S (2004).

7 Deng, Z. *et al.* Systematic analysis of trans and conjugated linoleic acids in the milk and meat of ruminants in *Advances in Conjugated Linoleic Acid Research, Volume 3* 45-93 (AOCS Publishing, 2006).

8 Destaillats, F., Trottier, J. P., Galvez, J. M. & Angers, P. Analysis of alpha-linolenic acid biohydrogenation intermediates in milk fat with emphasis on conjugated linolenic acids. *Journal of Dairy Science* **88**, 3231-3239, doi:10.3168/jds.S0022-0302(05)73006-X (2005).

9 Contarini, G., Povolo, M., Pelizzola, V., Monti, L. & Lercker, G. Interlaboratory evaluation of milk fatty acid composition by using different GC operating conditions. *Journal of Food Composition and Analysis* **32**, 131-140, doi:http://dx.doi.org/10.1016/j.jfca.2013.08.008 (2013).
